# Factors Associated with Health-Related Quality of Life in Mexican Lupus Patients Using the LupusQol

**DOI:** 10.1371/journal.pone.0170209

**Published:** 2017-01-23

**Authors:** I. Etchegaray-Morales, S. Méndez-Martínez, C. Jiménez-Hernández, C. Mendoza-Pinto, N. E. Alonso-García, A. Montiel-Jarquín, A. López-Colombo, A. García-Villaseñor, M. H. Cardiel, M. García-Carrasco

**Affiliations:** 1 Physiotherapy Program, Medical School, Benemérita Universidad Autónoma de Puebla, Puebla, México; 2 Systemic Autoimmune Diseases Research Unit, Regional General Hospital #36, Instituto Mexicano del Seguro Social, Puebla, México; 3 Puebla Research Coordination, Instituto Mexicano del Seguro Social, Puebla, México; 4 Department of Rheumatology, Benemérita Universidad Autónoma de Puebla, Puebla, México; 5 Department of Psychology, Medical School, Benemérita Universidad Autónoma de Puebla, Puebla, México; 6 Division of Health Research Highly Specialized Medical Unit, Traumatology and Orthopaedics Hospital, Instituto Mexicano del Seguro Social, Puebla, México; 7 Highly Specialized Medical Unit, Speciality Hospital of Puebla, Instituto Mexicano del Seguro Social, Puebla, México; 8 Medical School, Universidad de las Américas Puebla, Puebla, México; 9 Clinical Research Centre of Morelia, Michoacán, México; 10 Department of Immunology, Benemérita Universidad Autónoma de Puebla, Puebla, México; Peking University First Hospital, CHINA

## Abstract

**Introduction:**

Health-related quality of life (HRQOL) is affected by numerous clinical variables, including disease activity, damage, fibromyalgia, depression and anxiety. However, these associations have not yet been described in Mexican patients with systemic lupus erythematosus (SLE).

**Objective:**

To evaluate the relationship between disease activity, damage, depression and fibromyalgia and HRQOL measured by the LupusQoL-instrument in Mexican patients with SLE.

**Methods:**

A cross-sectional study was conducted in women fulfilling the 1997 ACR classification criteria for SLE. HRQOL was evaluated using a disease-specific instrument for SLE, the LupusQoL (validated for the Spanish-speaking population). Patients were evaluated clinically to determine the degree of disease activity and damage using the Mexican Systemic Lupus Erythematosus Disease Activity Index (Mex-SLEDAI) and Systemic Lupus International Collaborating Clinics-Damage Index (SLICC), respectively. Fibromyalgia and depression were assessed using the ACR criteria and the CES-D scale, respectively. The relationship between HRQOL and these variables was measured using Spearman’s rank correlation coefficient and linear regression analysis.

**Results:**

A total of 138 women with SLE, age 40.3±11 years, disease duration 8.8±6.4 years, with disease activity in 51.4%, depression in 50%, damage in 43% and fibromyalgia in 19.6% were included. Poorer HRQOL correlated with depression (r = -0.61; p< 0.005), fibromyalgia (r = -0.42; p< 0.005), disease activity (r = -0.37; p < 0.005) and damage (r = -0.31; p < 0.005). In the multivariate linear regression analysis, damage (β = -3.756, p<0.005), fibromyalgia (β = -0.920, p<0.005), depression (β = -0.911, p<0.005) and disease activity (β = -0.911, p<0.005) were associated with poor HRQOL.

**Conclusion:**

SLE disease activity, damage, fibromyalgia and depression were associated with poor HRQOL in our sample of Mexican SLE patients.

## Introduction

Systemic lupus erythematosus (SLE) is an autoimmune disease characterized by a complex pathogenesis with episodes of relapses and remissions [1]. SLE can cause substantial organic and functional disability, including debilitating fatigue, cognitive impairment, chronic renal disease and severe articular involvement [2]. Recent advances in medical care have considerably extended life expectancy in patients with SLE [3], although it remains lower than that of the general population, especially in patients with lupus nephritis [4]. In addition, some existing therapies are ineffective and poorly tolerated in a high proportion of SLE patients [2], and this has a substantial negative impact on the quality of life.

Disease-specific HRQOL is a multidimensional concept that represents the patient's general perception of the impact of the disease and/or treatment on their health. Various generic and disease-specific HRQOL instruments are available, but require rigorous methodological criteria to adapt them to different languages and, preferably, cultures. Generic instruments are applicable for various conditions, even in the absence of disease [5–7], but may need to be complemented by disease-specific instruments [3] that include domains such as sexual well-being, body image, fatigue, and independence. The LupusQol scale is a disease-specific patient-reported outcome measure validated in various languages that evaluates the quality of life in SLE patients [8].

HRQOL is reduced in patients with SLE compared with patients with acquired immune deficiency syndrome (AIDS), Sjögren’s syndrome, rheumatoid arthritis and with the general population [9, 10]. The incidence, prevalence and clinical evolution of SLE have been associated with ethnicity, socioeconomic status and environmental exposures. For example, poor social support is associated with high disease activity and poor mental functioning [2]. Misperceptions of the disease and mood disorders caused by poor quality of life in SLE patients worsen the prognosis and treatment adherence [11]. Therefore, assessment of the health status in SLE patients should include lupus disease activity, damage and HRQOL [12, 13, 14].

Cross-sectional and longitudinal studies, mainly using the LupusQoL-US and the LupusQoL, have demonstrated that numerous clinical variables, including disease activity, accumulated damage, fibromyalgia, depression and anxiety affect HRQOL in patients with SLE, with a high prevalence of depression and anxiety [1, 7, 8, 12–20]. Other studies have confirmed a low quality of life in SLE patients related to psychological alterations, especially depression and anxiety, body image, sleep disorders, planning, sexual relations and leisure activities with family and friends [21]. In addition, SLE patients present difficulties related to the activities of daily living and work performance, even compared with patients with other chronic diseases [22–24]. SLE patients also report severe fatigue, depressed mood and impaired HRQOL in addition to widespread pain and joint pain [25].

The reported prevalence of depressive symptoms in SLE varies between 17 and 71%, and may be due to the effects of medical treatment, psychosocial problems related to chronic disease or the disease itself [26, 27]. Studies show that increased disease activity, disease severity or a long disease duration increases vulnerability to depression. Shen et al studied 170 SLE patients and 210 healthy individuals and found that depression was the major contributor to worse HRQOL (ß = -0.616, p<0.05) [27]. Patients with more depressive symptoms were more likely to experience work disability [22].

HRQOL in patients with SLE and fibromyalgia is even lower [28, 29]. Patients with fibromyalgia report depression, pain and worse physical functioning.

In Mexico, a high prevalence of low HRQOL related to rheumatologic diseases, including SLE, rheumatoid arthritis and ankylosing spondylitis has been described. It has been shown that relatives and families tend to underestimate the effects on HRQOL compared with that perceived by patients themselves, and this may lead to problems in relating to others, communication and social support in patients with SLE [30]. García-Carrasco et al found that HRQOL, as measured by the SF-36 and by the LupusQol, was low in Mexican patients with SLE. The main domains affected were fatigue and burden to others in the LupusQoL and vitality, bodily pain and physical component summary in the SF-36 [12].

The correlations between HRQOL and disease activity, damage, fibromyalgia and depression have not been studied in the Mexican SLE population. Therefore, due to the clinical relevance of HRQOL in health disparities and severity of SLE, the aim of this study was to determine whether disease activity, damage, fibromyalgia, and depression were associated with HRQOL in Mexican women with SLE.

## Materials and Methods

### Disease activity

In the first interview, all patients who fulfilled the 1997 ACR classification criteria for SLE [31] were evaluated using the Mexican Systemic Lupus Erythematosus Disease Activity Index (Mex-SLEDAI), which contains 10 major clinical and laboratory variables. The Mex-SLEDAI is a validated instrument that does not include immunologic measures such as anti-DNA or complement levels [32]. It measures disease activity in the last 10 days with a sensitivity of 85.7% and a specificity of 100%. The Mex-SLEDAI has scores from 0 to 24. A score of 0–1 represents no disease activity and a score of ≥ 2 represents disease activity [33].

Marital status was defined as: married, living with a partner, single, divorced and widowed. For the statistical analysis, the data were divided into two variables: patients not being living with a partner or spouse (single, divorced and widowed) and those living with a partner or married [34]. Written informed consent was obtained from all patients. The Local Committee of Health Investigation # 2102 of the Mexican Institute of Social Security approved the study.

### Damage

The Systemic Lupus International Collaborative Clinics /American College of Rheumatology Damage Index (SLICC/ACR Damage Index) developed by the SLICC/ACR group is an instrument validated to assess damage in SLE patients and quantifies accumulated damage due to disease or treatment when present for ≥ 6 months. It includes 41 items covering 12 organ systems: eye, neuropsychiatric, renal, pulmonary, cardiovascular, peripheral vascular, gastrointestinal, musculoskeletal, skin, premature ovarian failure, metabolism and neoplasm. Damage is diagnosed if the score is ≥1. This instrument has an intraclass correlation coefficient of 0.553 [35].

### Fibromyalgia

Fibromyalgia was assessed and classified according to the 2010 ACR criteria by a rheumatologist specializing in fibromyalgia before data were collected for the present study. The ACR criteria include two concomitant items: (1) chronic generalized pain in both sides of the body, both axial and peripheral, below and above the waist; (2) the presence of ≥ 11 of the 18 specified tender points described for fibromyalgia when a uniform pressure of 4 kg is applied with the thumb. When palpation is painful the zone is considered highly-sensitive [36–38]. Fibromyalgia is diagnosed when the two criteria are demonstrated. For statistical analysis, patients were divided into two categories: fibromyalgia yes/no. Fibromyalgia can be identified from other rheumatic conditions by the use of simple criteria with good sensitivity (88.4%), specificity (81.1%) and accuracy (84.9%) [38].

### Depression

The validated Center for Epidemiological Studies Depression Scale (CES-D), which contains 20 items rated on a Likert scale from 0 (never or almost never) to 3 (most or all the time) and measures symptoms during the last week, with a range of 0–60 (higher scores reflect increased symptom severity), was used to measure depression. Item responses range from 0 to 3, where 0 = rarely or none of the time (less than 1 day in the past week), 1 = some or a little of the time (1–2 days), 2 = occasionally or a moderate amount of the time (3–4 days), and 3 = most or all of the time (5–7 days). The CES-D also provides cutoff scores (e.g. ≥16) that help identify individuals at risk for clinical depression [39]. The scale is reliable (r = 0.88), has internal consistency (alpha coefficients ranging from 0.80 to 0.90), moderate test-retest reliability (r = 0.40 and higher), and high concurrent and construct validity [40].

### Health-related quality of life

HRQOL was measured using the validated Spanish-language version of the disease-specific LupusQoL, developed in the UK [8]. It has 34 items in eight domains with individual subscales: physical health (8 items), emotional health (6 items), body image (5 items), fatigue (4 items), pain (3 items), planning (3 items), burden to others (3 items), and intimate relationships (2 items). The physical health, future planning and pain domains reflect present and future activities and how these are modified by SLE. The emotional health and fatigue domains reflect aspects related to the emotional state, sleep quality and vitality. The body image domain reflects how SLE affects the patient’s view of their body. The burden to others domain reflects the patient’s perception of how SLE makes them a burden to others. The intimate relationships domain reflects how SLE affects sexual relations [41]. The questionnaire is measured on a 5-point Likert scale (all the time = 0, most of the time = 1, a good bit of the time = 2, occasionally = 3 and never = 4) with a recall period of items of 4 weeks. The mean raw domain score is transformed to scores ranging from 0 (worst HRQOL) to 100 (best HRQOL) by dividing by four and then multiplying by 100. The mean raw domain score is then calculated by totalling the item response score of the items and dividing by the number of items. The LupusQoL has good internal consistency (Cronbach’s alpha 0.88 to 0.95) and good test-retest reliability (r 0.72 to 0.93), good concurrent validity with comparable domains of the SF-36 (r 0.71 to 0.79) and has discriminative validity for different levels of disease activity measured by the British Isles Lupus Assessment Group (BILAG) index and damage (SLICC/American College of Rheumatology (ACR)-Damage Index (DI)), but not for all domains: the LupusQoL domains that correlate with the SLICC/ACR DI are physical health, pain, planning, intimate relationships and burden to others, but not emotional health, body image and fatigue [8].

### Statistical analysis

Descriptive statistics were made using means, standard deviation and percentages. The relationship between disease-specific HRQOL and disease activity, damage, fibromyalgia and depression was evaluated using Spearman´s rho. After the bivariate analysis, significant correlations were included in a multivariate linear regression analysis. All tests were two-tailed, with a p value<0.05 considered statistically significant. SPSS version 21 (Chicago, Illinois, USA) for Windows XP software was used to analyse the data.

## Results

### Subjects and quality of life

**Fig 1:**
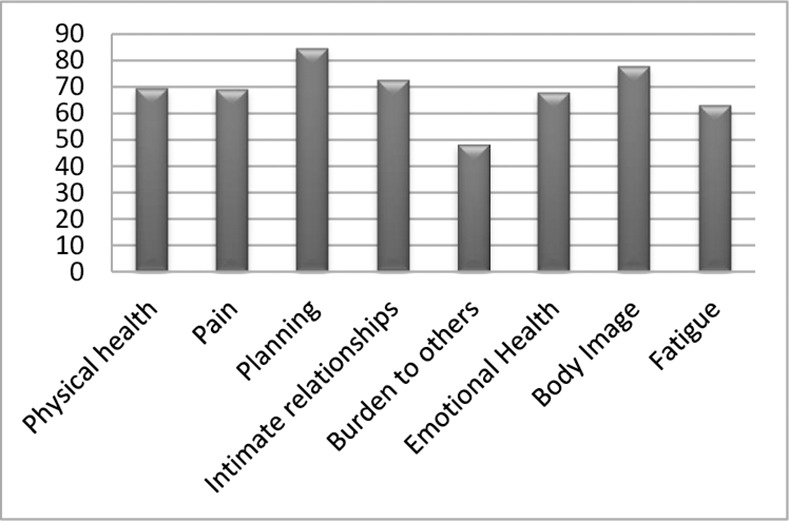
Mean LupusQoL domain scores in patients with SLE.

A total of 138 women with SLE, mean age 40.3 ± 11 years, mean disease duration 8.8 ± 6.4 years, were included: 48.6% had mild-to-moderate disease activity, mean 2.3 ± 2.9 years, and 43.5% had damage, mean 0.72 ± 1, within the range shown by other studies [42, 43], while 19.6% met the criteria for the diagnosis of fibromyalgia: tender points were 4.9 ± 5.9 high sensitivity points/zones. Mean depression score was 16.1 ± 1, (Table 1). Mean HRQOL was 68.4 ± 21 on the LupusQol. The domains with the worst scores were burden to others, fatigue, and emotional health, while the planning domain had the best score (Fig 1). The mean LupusQol score was 51 ± 24 in patients with fibromyalgia and 72 ± 18 in those without. Fifty percent of patients had depression according to the CESD-R. In the multiple regression, depression was not associated with disease activity (rho = 0.163, p = 0.056) or damage (rho = 0.117, p = 0.171) but was associated with fibromyalgia (rho = 0.241, p = 0.04).

**Table 1:** Sociodemographic, clinical, HRQOL and specific domain characteristics of patients with systemic lupus erythematosus.

Variable	N = 138
Age, mean ±SD, years	40.3 ± 11 (19–73)
Disease duration, mean ±SD, years	8.8 ± 6.4 (0–31)
Disease activity, mean ±SD, score	2.3 ± 2.9 (0–13)
Damage, mean ± SD, score	0.72 ± 1 (0–5)
Fibromyalgia points, mean ±SD, score	4.9 ± 5.9
Depression CES-D, mean ±SD, score	16.1 ± 1 (0–48)

There were significant correlations (Spearman’s rho p <0.005) between disease activity, damage, depression and fibromyalgia and all disease-specific HRQOL domains. Age correlated negatively with body image (r = -0.221, p = 0.009) and pain (r = -0.206, p = 0.015). Educational level, socioeconomic status, disease duration and steroid doses were not associated with HRQOL domains.

### Variables associated with poor HRQOL

HRQOL was 61.8 ± 21.1 in patients with damage versus 73.5 ± 20.3 in those without (p = 0.001). Patients with disease activity had a mean HRQOL of 59.9 ± 21.7 versus 76.5 ± 17.8 in those without (p = 0.005). HRQOL was 53.4 ± 22.8 in patients with fibromyalgia and 72.1 ± 19.5 in those without (p = 0.005). Mean HRQOL was 57.1 ± 19.4 in patients with depression and 79.8 ± 17 in those without (p = 0.005). Marital status was not associated with poor HRQOL (p = 0.399). The most affected domains were: burden to others, fatigue and the psychological domain. Depression (r = -0.61, p< 0.005), fibromyalgia (r = -0.42, p< 0.005), disease activity (r = -0.37, p< 0.005) and damage (r = -0.31, p < 0.005), correlated negatively with poorer quality of life and were included in the linear regression analysis, which showed that damage β -3.75 (95% CI -6.367/-1.146) (p < 0.005), fibromyalgia β -0.92 (95% CI -1.406/ -0.435) (p < 0.005), disease activity β -0.91 (95% CI -1.884/0.63) (p <0.005) and depression β -0.91 (95% CI -1.158/-0.664) (p < 0.005) worsened HRQOL (Tables 2 and 3).

**Table 2:** Correlation between HRQOL and clinical parameters.

Spearman Rank correlation	
Clinical parameters	r values (95% CI)
Depression	-0.61(-0.72 to -0.51)*
Disease activity	-0.37(-0.51 to -0.21)*
Damage	-0.31(-0.46 to -0.17)*

*p = 0.005.

**Table 3:** Multiple-linear regression analysis of factors associated with poor HQOL.

Coefficients^a^								
Model		Non-standardized coefficients		Standardized coefficients	T	Sig.	95% confidence intervals of beta	
		B	Standard error	Beta			Upper limit	Lower limit
1	(Constant)	92.674	2.553		36.298	.000	87.624	97.724
	Damage	-3.756	1.320	-.184	-2.846	.005	-6.367	-1.146
	Fibromyalgia	-0.920	.246	-.253	-3.748	.000	-1.406	-.435
	Activity	-0.911	.492	-.124	-1.850	.005	-1.884	.063
	Depression	-0.911	.125	-.473	-7.294	.000	-1.158	-.664

a. Dependent variable: Results

## Discussion

Disease-specific HRQOL plays an important role in the evaluation of patients with SLE. The LupusQoL scores of our patients were generally good, except for the burden to others domain. Some studies have reported that, in SLE patients, HRQOL is worsened by disease activity. Other studies have reported that depression is an important predictor of low HRQOL in SLE patients [18, 27]. Our study confirms these results, showing that SLE patients with active disease and/or depression had worse HRQOL scores. The components most affected in our patients were: burden to others, fatigue and emotional health, similar to the results of other studies [3, 7, 19]. Disease activity, damage, fibromyalgia and depression all negatively affected HRQOL, although disease activity and damage was low, similar to that reported by most studies [3, 7, 13, 16].

Our results emphasize the statement by McElhone et al suggesting that disease-specific HRQOL is a different entity from disease activity and damage, and therefore the three aspects should be viewed as independent entities in SLE patients in order to obtain the complete clinical context [10].

The frequency of fibromyalgia in our study was lower than the 35.7% reported by Torrente-Segarra et al [44] but higher than that found by Araújo et al [45], who stated that fibromyalgia in SLE patients has a strong impact on HRQOL. Valencia-Flores et al found a rate of fibromyalgia of 9.5% in SLE patients, significantly lower than the 19.6% in patients with similar disease activity but older age and longer disease duration found in our study [37].

Fifty percent of our sample had depression, reflecting other studies that found depression was a significant predictor of poor HRQOL [26, 27].

Mean disease-specific HRQOL scores were lower in patients with greater disease activity, greater damage, fibromyalgia and depression. Additional studies including patients with greater disease activity and damage are required to identify whether these factors contribute to poor disease-specific HRQOL in our setting and to create strategies that could improve disease-specific HRQOL in SLE patients with fibromyalgia. Like McElhone et al, we found no significant differences in LupusQoL scores according to the marital status [34].

The clinical importance of our results is that the factors associated with poorer HRQOL, such as depression, disease activity and fibromyalgia facilitate an integrated vision of the suffering of these patients and the detection of potentially-modifiable aspects that could improve treatment compared with an isolated view of the disease and HRQOL as separate entities.

The main limitation of our study was that it was cross-sectional and conducted in a single centre. However, we believe that these findings should be considered suggestive and not conclusive. Another limitation is that most patients had low levels of activity and therefore the results cannot be extrapolated to patients with higher levels of disease activity, which could have greater effects on the quality of life. Likewise, other variables, such as renal impairment, sleep disturbances, cognitive dysfunction, socioeconomic status, personality traits, coping, social support, anxiety, abnormal laboratory results and medications were not assessed, and these could impact disease-specific HRQOL and appear to play an important role in the disease process.

In conclusion, we found that the main risk factor associated with worse HRQOL in Mexican patients with SLE is depression, followed by fibromyalgia and damage. These findings might be used to implement correct surveillance of these factors and establish management programs that could improve the quality of life in these patients.
